# Detection of *Leishmania* metacyclogenesis within the sand fly vector employing a real-time PCR for *sherp* gene expression: A tool for *Leishmania* surveillance and transmission potential

**DOI:** 10.1371/journal.pntd.0012915

**Published:** 2025-03-17

**Authors:** Chukwunonso O. Nzelu, Somayeh Bahrami, Phillip G. Lawyer, Nathan C. Peters

**Affiliations:** 1 Department of Microbiology, Immunology, and Infectious Diseases, Cumming School of Medicine, and Faculty of Veterinary Medicine, Snyder Institute for Chronic Diseases, University of Calgary, Calgary, Alberta, Canada; 2 Department of Parasitology, Faculty of Veterinary Medicine, Shahid Chamran University of Ahvaz, Ahvaz, Iran; 3 Monte L. Bean Life Science Museum, Brigham Young University, MLBM, Provo, Utah, United States of America; Wadsworth Center, UNITED STATES OF AMERICA

## Abstract

Surveillance of infected insect vectors of vector-transmitted diseases has been recognized for its ability to estimate pathogen prevalence and transmission potential. Classically restricted to microscopic dissection and examination of individual insects, the potential of entomological monitoring has grown due to the advent of rapid molecular DNA detection methods with high specificity and sensitivity. Despite such advancement, a recurring question concerning DNA detection of parasitic pathogens is related to the fact that DNA amplification, by itself, does not differentiate between insects carrying infectious versus dead, non- or poorly-infectious life-cycle stages, thereby limiting it’s programmatic usefulness for accurately measuring the transmission potential of infected insects in endemic areas or within experimentally infected populations. Herein, we developed a quantitative real-time PCR with Reverse Transcription (RT-qPCR) based *sherp* (small hydrophilic endoplasmic reticulum-associated protein) detection assay employing a novel set of *sherp*-RT-qPCR primers to detect and quantify infectious *Leishmania* parasites in infected vector sand flies. The *sherp* RT-qPCR showed significantly increased expression of *sherp* transcripts in infectious *Leishmania* metacyclic versus non-metacyclic promastigotes or mammalian-derived amastigotes. The assay displayed detection performance ranging from 10^6^ to 1 parasite and could reliably quantify parasites within infected sand flies without the need for dissection. *Sherp* transcripts were also successfully amplified from flies stored in ethanol at room temperature, a practical and economical method of sample preservation in resource-limited field settings. Lastly, in conjunction with an established RT-qPCR assay for *Leishmania* kinetoplast DNA minicircles, we were able to calculate a score for the degree of metacyclogenesis within infected sand flies, a known predictor of transmission potential. These results highlight the potential of the *sherp*-RT-qPCR assay to identify hotspots of potential transmission, areas of re-emergence, vector competence, and the transmission potential of infected sand fly populations.

## Author summary

Monitoring vector populations for vector-transmitted pathogens is one means by which to survey endemic areas for potential transmission, hotspots of infection intensity, and pathogen prevalence. In the case of many parasitic pathogens, this is a labor-intensive endeavor and is complicated by the fact that insect vectors can carry multiple parasite life-cycle stages, only some of which are infectious. During *Leishmania* infection of the sand fly vector, the parasite undergoes a maturation process in the gut of infected flies over time and only mature infections, as indicated by high numbers of the metacyclic promastigote life cycle stage, are associated with efficient transmission and enhanced disease. Therefore, developing a large-scale, field-adaptable surveillance assay is advantageous to determine the prevalence of infected sand flies with mature infections. Here, we report a molecular methodology to identify infected sand flies with mature infections based on the expression of a metacyclic promastigote associate gene, *sherp*. This assay could be applied in field and laboratory settings to determine pathogen prevalence, vector competence, and transmission potential.

## Introduction

Leishmaniasis is a vector-borne parasitic disease caused by more than 20 species of the obligate intracellular protozoan parasite of the *Leishmania* genus and is transmitted by the bite of an infected female sand fly. The disease is endemic in 98 countries, affecting 12 million people worldwide, with an estimated 700,000 to 1 million new cases occurring annually [1]. After deposition into the skin, *Leishmania* can give rise to a spectrum of clinical manifestations that are largely associated with the infecting strain of the parasite and the host immune response [1]. The spread of leishmaniasis depends on the distribution of the sand fly vector. Approximately 1000 sand fly species have been described, but only species belonging to the genus *Phlebotomus* in the Old World and *Lutzomyia* in the New World are medically important [2–4].

The life cycle of *Leishmania* parasites is digenetic, alternating between fusiform motile extracellular promastigotes that develop in the alimentary tract of the sand fly vector and spherical immobile intracellular amastigotes that reside in mammalian phagocytic cells. Within infected sand flies parasites undergo a bifurcated developmental process known as metacyclogenesis that generates a series of morphological and functional differentiation stages including non- or poorly-infectious forms and highly infectious metacyclic promastigotes [5]. Metacyclogenesis in *Leishmania* can be induced *in vitro* by low pH, low tetrahydrobiopterin levels, and nutrient depletion [6], and correlates with stationary phase growth and a general down-regulation of synthetic activity [7]. The triggers for metacyclogenesis in the sand fly are likely to be more complex and are significantly influenced by the sand fly gut microbiota and sucrose levels [8,9]. The main promastigote forms within the sand fly gut are procyclics, nectomonads, leptomonads, haptomonads, and metacyclics [10,11]. More recently, a new form called retroleptomonads, which originate from retained metacyclics after a subsequent blood meal by the infected sand fly, have been observed [12]. Unlike other stages of promastigotes, midgut-detached metacyclic promastigotes are found in the anterior midgut near the stomodeal valve of the sand fly [13], and display distinctive morphological and biochemical features (small cell body and relatively long flagellum, highly motile and resistant to human complement), which facilitates the parasite survival in the mammalian host following transmission [11]. The presence of metacyclic promastigotes in the infectious inoculum is a critical determinant of transmission potential, the establishment of infection, and infection outcome [14,15].

Several genes, such as *hasp* (hydrophilic acylated surface protein), *sherp* (small hydrophilic endoplasmic reticulum-associated protein), the glycoprotein 63 (*gp63*) metalloprotease family, and autophagy genes have been identified to be specifically expressed in the *Leishmania* late stages of metacyclogenesis [5,16–20]. Moreover, the *Leishmania*-specific *sherp* (6.2 kDa and acidic pI of 4.6) gene is one of the best-characterized genes associated with metacyclogenesis, is encoded on chromosome 23 (cDNA16 locus), and is highly conserved within the *Leishmania* genus [19–22]. Although *sherp* is detectable in other *Leishmania* stages, the *sherp* transcript is highly up-regulated in infective metacyclic parasites [5,15,19,21], where it localizes to the cytosolic faces of the mitochondrion and endoplasmic reticulum [23,24]. The *sherp*-coded protein has been shown to play an important role in *Leishmania* metacyclogenesis and parasite transmission in the sand fly and the establishment of infection in the host [15,19]. It has also been shown to be up-regulated in sand fly-derived promastigotes versus cultured promastigotes [20]. *Sherp* deficiency results in stalled metacyclogenesis in sand fly-derived promastigotes compared to culture promastigotes and supports its key role in successful metacyclogenesis in the sand fly gut versus culture [19].

Hitherto, detection of *Leishmania* parasites within individual sand flies relied largely on dissection and microscopic examination of individual flies, which is technically demanding, laborious, and time-consuming, especially when large numbers of specimens must be examined due to the low *Leishmania* infection rate in sand flies (0.01% - 1%), even in endemic areas [25–27]. To overcome these technical limitations, in the last three decades, molecular approaches (PCR formats) have been increasingly employed in the detection of *Leishmania* DNA in individual or pooled sand flies [26–28]. However, one of the difficulties in applying a DNA PCR detection/quantification assay to monitor actual transmission potential versus simply the presence of the parasite is distinguishing sand flies carrying infections with no or low levels of metacyclogenesis from those carrying the infectious metacyclic stage. The ability to do this is what distinguishes basic molecular xenomonitoring, the screening of haematophagous insects for the presence of a pathogen’s genetic material, from actual entomological monitoring of transmission potential.

Furthermore, the dose of infecting parasites within sand flies can influence the number of differentiated infective-metacyclic promastigotes, the parasite transmission potential to the host, and the severity of infections [29]. For instance, in an experimental model, low-dose infected sand flies gave rise to infections with low parasite numbers and low frequencies of metacyclic promastigotes and transmitted less severe diseases, albeit with significant reservoir potential [14,29]. In contrast, high-dose infected sand flies were enriched for metacyclic forms and resulted in higher transmission efficiencies and increased disease severity in the mammalian host [14,29,30]. Although there is no published data on the frequency/number of metacyclic promastigotes within infected field-caught flies so far as we are aware, the composition of the transmitted dose is a crucial determinant of disease outcome and onward transmission in experimental models and these low versus high dose experimental scenarios might have implications in leishmaniasis endemic and outbreak foci. In addition, transmission efficiency in the field and/or experimental models may also be complicated by the presence of varying promastigote forms in the sand fly gut, as no infected fly transmits a homogenous population of metacyclics [5,14,15]. The prediction of the degree of transmission competence within a group of naturally or experimentally infected flies will rely on how the flies are enriched for metacyclics versus other promastigote forms. Therefore, mass screening of sand flies for *Leishmania* metacyclogenesis in a surveillance strategy has the potential to provide an accurate assessment of not only the presence or absence of the parasite but also transmission potential in endemic areas and highlight hotspot areas of potential transmission.

Recognizing these operational gaps, we developed and standardized a quantitative real-time PCR with reverse transcription (RT-qPCR) assay based on the *sherp* gene to determine the transmission potential of infected sand flies for use in field and laboratory settings.

## Methods

### Ethics statement

All animal experiments were performed under a study protocol approved by the University of Calgary Animal Care Committee (Protocol # AC14-0142 and AC19-007) in compliance with the Canadian Council for Animal Care Guidelines.

### Mice

Six-week-old female C57BL/6J (WT) mice were purchased from The Jackson Laboratory and maintained under specific pathogen-free conditions at the University of Calgary, Cumming School of Medicine, Animal Resource Centre.

### Parasites and isolation of metacyclic promastigotes

Experiments were performed using *Leishmania* (*Leishmania*) *major* (MHOM/IL80/Friedlin) or *Leishmania* (*L.*) *major* (RYN, a derivative of WR2885), *Leishmania* (*L.*) *infantum* (MHOM/ES/92/LLM-320) and *Leishmania* (*L*.) *donovani* (MHOM/IN/83/Mongi-142). Promastigotes were grown *in vitro* at 26 ^◦^C in complete medium 199 (M199), supplemented as follows: 20% heat-inactivated FCS (Sigma-Aldrich), 100 U/mL penicillin, 100 μg/mL streptomycin, 2 mM L-glutamine, 40 mM Hepes, 0.1 mM adenine (in 50 mM Hepes), 5 mg/ml hemin (in 50% triethanolamine), and 1 mg/mL 6-biotin. Infective metacyclic promastigotes and non-metacyclic parasites were purified in a Ficoll gradient [31].

### Sand fly infection and follow-up

*Lutzomyia* (*Lu*.) *longipalpis* (Jacobina, Brazil) was obtained from the Walter Reed Army Institute of Research (WRAIR) via BEI Resources, Bethesda, Maryland, USA, and maintained at the sand fly insectary in the Peters’ Laboratory at the University of Calgary, Calgary, Canada [32]. Recently, *Lu. longipalpis* has been shown to be a competent vector for *L.* (*L.*) *major* [33]. For sand fly infections, five-day-old female sand flies were infected with promastigotes of *L.* (*L.*) *major* (RYN) washed twice in PBS and resuspended in heparinized, heat-activated pooled mouse blood through an artificial ethanol-sterilized chick-skin membrane feeding system at a density of 2 x 10^6^ amastigotes per ml [14]. Blood-fed flies were separated and kept on a 30% sucrose diet under 26 ^◦^C and 80% RH. Flies were denied from laying eggs to minimize post-oviposition mortality. At 4-, 7-, and 14 days post-infection, sand flies were collected to assess the infection status and for downstream RT-qPCR assays. Briefly, for midgut dissection, flies per experimental group were anesthetized with CO_2_, killed in 5% soap solution, and midguts were dissected and transferred into tubes containing 50μl of complete M199 medium. The guts were macerated briefly using a plastic pestle, a 10μl sample was counted under a hemocytometer, and the number of metacyclic promastigotes was determined by morphology and movement. All dissected flies with mature infections were observed to contain eggs. In addition, whole infected flies were collected at 4-, 7-, and 14- days post-infection, preserved in absolute ethanol, and stored at room temperature (2-4 or 14- months) until used for RT-qPCR assays. Previous observations suggest that a second blood meal in flies with retained parasites can result in larger infections with a higher proportion of infective metacyclic promastigotes [12]. In some experiments, experimentally infected sand flies were allowed to acquire a second blood meal via exposure to naïve mice on d14 p.i. and RT-qPCR was performed to assess the presence of metacyclic promastigotes following an additional 7 days on a 30% sucrose diet at 26 ^◦^C and 80% RH (21 total days post-initial sand fly infection).

### Experimental transmission of *Leishmania* via sand fly bites or needle challenge

For transmission by sand fly bite, four C57BL/6J (WT) mice were anesthetized by intraperitoneal (i.p.) injection of 30 μl of ketamine/xylazine (100 mg/mL). The mice bodies were screened with Gauze Sponges (non-woven) (Technologist Choice, Dis-022B) except their ears and placed into a custom-made polycarbonate adult holding cage (30 x 30 x 30 cm) containing 200-300 day 14 post-infection female *Lu. longipalpis* flies, starved overnight. Infected flies were allowed to feed ab-libitum on exposed ears for 45-90 minutes. Feeding was monitored to ensure equivalent exposure between individual ears, and following exposure, ears were examined for hematoma in the skin to confirm that biting occurred on all ears.

For infection by needle inoculation, six-to 8-week-old female C57BL/6J (WT) mice were infected by intra-dermal injection of the dorsal surface of both ears with 2 x 10^5^ *L.* (*L.*) *major* (MHOM/IL80/Friedlin) metacyclic promastigotes in a volume of 10 μl employing an insulin needle (BD Biosciences). At 48 hours post-transmission, mice were humanely euthanized, and the ears were processed and subjected to RT-qPCR assays.

### Post-transmission follow-up and Experimental endpoints

Animals were monitored weekly to follow the development of lesions caused by *L*. (*L*.) *major* infection by measuring ear lesions with a Digimatic Vernier calipers (Mitutoyo Corp.). All animals were humanely euthanized at 8 weeks post-infection.

### Processing of tissue ears

Ears were removed and placed in 70% ethanol for 2-5 minutes and then allowed to dry. The ventral and dorsal sheets of the ear were separated and incubated in DMEM containing 160 μg/mL of Liberase (Roche Diagnostic) for 90 minutes at 37 ^◦^C and 5% CO_2_. Digested ear sheets were homogenized for 3^1^/_2_ minutes in a Medicon/Medimachine tissue homogenizer system (Beckton Dickinson) and then flushed from the Medicon with 10 mL DMEM media containing 0.05% DNase I and filtered using a 50 μm-pore-size cell strainer.

### RNA extraction and quantitative reverse transcription PCR (RT-qPCR)

Ear cell homogenates, parasites isolated from stationary culture, dissected midguts, or whole flies homogenized in RLT Buffer (Qiagen) using a plastic pestle were passed through QIAshredder columns, RNeasy mini-Kit (Qiagen, Hilden, Germany). In some sample preparations, a DNase 1 (Sigma-Aldrich, USA) treatment step was included in addition to the recommended RNA isolation procedure to remove potential genomic DNA contamination from the total RNA preparation but this did not impact the results, suggesting RNA purification using the RNeasy mini-Kit (Qiagen efficiently removes potential genomic DNA contamination (by efficient on-column digestion of genomic DNA) (Qiagen). The mRNA concentration and purity were measured with a Nano-drop ND-1000 (Thermo Scientific). Reverse transcription was performed using the High-Capacity cDNA Reverse Transcriptional Kit (Thermo Fisher). For the RT-qPCR assay, a set of forward and reverse primers was designed based on the *Leishmania sherp* gene in the present study (Table 1). Real-time PCR was performed on an ABI Prism 7900 sequence detection system (Applied Biosystems), and the reaction was carried out in a final volume of 10 μl, containing 2 μl cDNA, a SYBR-Green detection system (Thermo Fisher), and 400 nM of each primer. The target *sherp* gene was normalized to the *Leishmania* 18S rRNA F 5’-GGGAAACCCCGGAATCACAT-3’and 18S rRNA R 5’-GGTGAACTTTCGGGCGGATA-3’ endogenous/reference control [34], and non-infected ear tissue, -dissected fly midguts or -whole sand flies. The results were analyzed by the comparative threshold cycle method using 2^−ΔΔCT^, in which the differences in Ct (cycle threshold) between target and reference genes are normalized to gene expression in a calibrator sample to determine the fold increase over gene expression in the uninfected (Naïve, NI) control group [35]. Serial 10-fold dilutions of *L.* (*L.*) *major* cDNA (1,000,000 (10^6^) to 1 (10^0^) parasite equivalents) from stationary phase culture-derived purified metacyclic promastigotes were used to establish a standard curve. Water only as well as cDNA from uninfected whole sand fly females were used as negative controls. Furthermore, to confirm the *sherp*-RT-qPCR *Leishmania* detection in whole flies screened, a highly sensitive *Leishmania* kinetoplast DNA minicircles region (*kDNA*) was used for the RT-qPCR target employing the primers (JW11: 5^′^-CCTATTTTACACCAACCCCCAGT-3^′^ and JW12: 5^′^ - GGGTAGGGGCGTTCTGCGAAA-3′) [36].

**Table 1:** RT-qPCR primers designed to target the *Leishmania sherp* gene in this study.

Primer name	Target gene	Primer sequence 5’- 3’
Le. SHERP-F (Forward)	SHERP	CTCGGCATACGCGGTCTCTC
Le. SHERP-R (Reverse)	SHERP	CTGCAGCCTTGTTCCCCACA

### Data representation and statistical analysis

Results shown are per sample derived from individual *Leishmania* cultures, dissected sand fly midguts, whole infected sand flies, or infected mouse ears, with a representation of the group mean value ± SD or SEM as indicated. The non-parametric Mann-Whitney test was used to determine the differences between two groups, and comparisons between multiple groups were done using a Kruskal-Wallis test with Dunn’s post-test to correct for multiple comparisons. *P ≤ 0.05, **P ≤ 0.005, ***P ≤ 0.0005, ****P ≤ 0.0001. The RT-qPCR efficiency was calculated from the linear regression standard curve using the equation: E = 10^−1/slope^ [37]. Linear regression, calculation of parasite number from a standard curve, and statistical analyses were conducted using GraphPad Prism version 10.

## Results

### Standardization and detection sensitivity of a *sherp* RT-qPCR assay for absolute quantification of *Leishmania* metacyclic promastigotes

**Fig 1:**
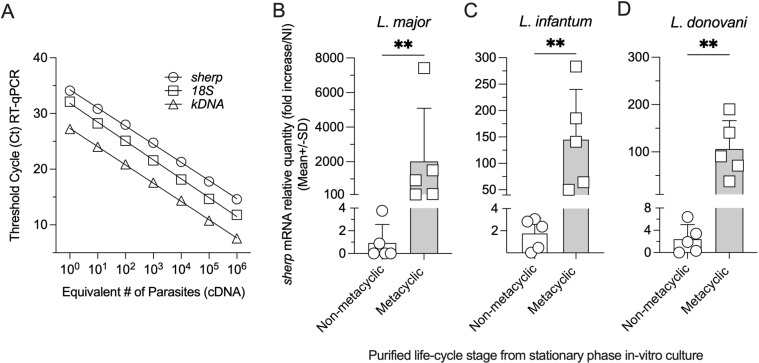
Detection and quantification of culture-derived *Leishmania* metacyclic promastigotes by RT-qPCR. (A) Ten-fold serial dilutions of cDNA prepared from purified metacyclic promastigotes of stationary phase cultures were used to generate a standard curve for each target gene. Each symbol presents a sample ranging from 10^6^ to 1 parasite equivalents for *sherp*, *18S* rRNA, and *kDNA* RT-qPCR targets. The RT-qPCR was run employing a single plate in a single RT-qPCR reaction. (B–D) Cultured-derived *L.* (*L*.) major, *L.* (*L*.) *infantum,* and *L*. (*L*.) *donovani* non-metacyclic and infectious metacyclic promastigotes (10^6^ parasite cells per lifecycle stage) were purified and assessed for *sherp* transcript expression using RT-qPCR. In **(B-D)** n=5 pooled individual experiments. **P=0.0079 employing a Mann-Whitney test.

To detect metacyclic promastigotes by employing RT-qPCR, we first designed a novel set of primers to amplify transcripts of the metacyclic-enriched *Leishmania sherp* gene [5,19–22]. A set of forward (Le. SHERP-F) and reverse (Le. SHERP-R) primers were designed manually based on the conserved region of the *Leishmania sherp* gene (GenBank accession no. AJ237587). The selected *sherp*-RT-qPCR primers are shown in Table 1. A Primer-Blast search, using the sequences of the *Leishmania sherp* oligonucleotides in the “Refseq mRNA” set of the NCBI database, revealed homologous identity with only *sherp* (XM_001683391, XM_001683388, XM_001683392) and had the highest degree of homology with *L.* (*L.*) *major* cDNA16 gene family (100%). The sensitivity of the broad range *sherp*-RT-qPCR was assessed by employing serial dilutions of *L.* (*L.*) *major* (MHOM/IL80/Friedlin) cDNA (10^6^ to 1 parasite equivalents) prepared from purified metacyclic promastigotes obtained from stationary phase cultures and compared with detection of *Leishmania* 18S rRNA [34] and a highly sensitive *Leishmania* kinetoplast minicircle (*kDNA*)-RT-qPCR [36] (Fig 1A). As expected, the *kDNA* primers were highly sensitive, requiring fewer cycles to detect the same number of metacyclic promastigotes versus 18S and *sherp* primers, likely due to the fact that there are thousands of copies per *Leishmania* parasite. In each case, the detection of *Leishmania* cDNA was linear from 10^6^ to 1 parasite equivalents. Under these conditions, it was possible to obtain a high coefficient of determination (R^2^ = 0.9994) and efficiency of 101.1% for *sherp*-RT-qPCR. The results indicate the efficiency of our *sherp* primer set to reliably detect and quantify *Leishmania* mRNA from purified metacyclic promastigotes. Due to its high sensitivity, the *kDNA*-RT-qPCR was subsequently used alongside our *sherp*-RT-qPCR for the confirmation of the detection and quantification of *Leishmania* in sand fly samples in the study.

It has been previously indicated that the use of primers designed to detect *sherp* expression in one *Leishmania* species or strain may not be applicable to other *Leishmania* species or strains [15]. To determine the specificity of our primer set for different *Leishmania* species and to discriminate between metacyclic and non-metacyclic parasites, we employed *sherp*-RT-qPCR to detect *sherp*-transcripts within non-metacyclic and infectious metacyclic promastigotes isolated from stationary phase cultures of *L*. (*L*.) *major*, *L*. (*L*.) *infantum*, and *L*. (*L*.) *donovani* employing Ficoll purification [31]. Employing the *sherp*-RT-qPCR assay, we detected high levels of the *sherp*-transcript within the purified metacyclic promastigote fraction of each *Leishmania* strain and low or undetectable levels in the non-metacyclic fraction, suggesting that the metacyclic promastigote lifecycle stage of *Leishmania* is highly enriched for *sherp* expression and that our *sherp*-RT-qPCR primer set is able to detect *sherp*-expression in multiple *Leishmania* species (Fig 1B–D). Previous studies have suggested that interspecies comparisons employing *sherp* expression require careful optimization for each species, and we also suggest appropriate species-specific validation is likely to be required for the *sherp*-RT-qPCR employed here before application to other *Leishmania* species [15,20].

### Quantification of *sherp*-expression within infected sand fly midguts

**Fig 2:**
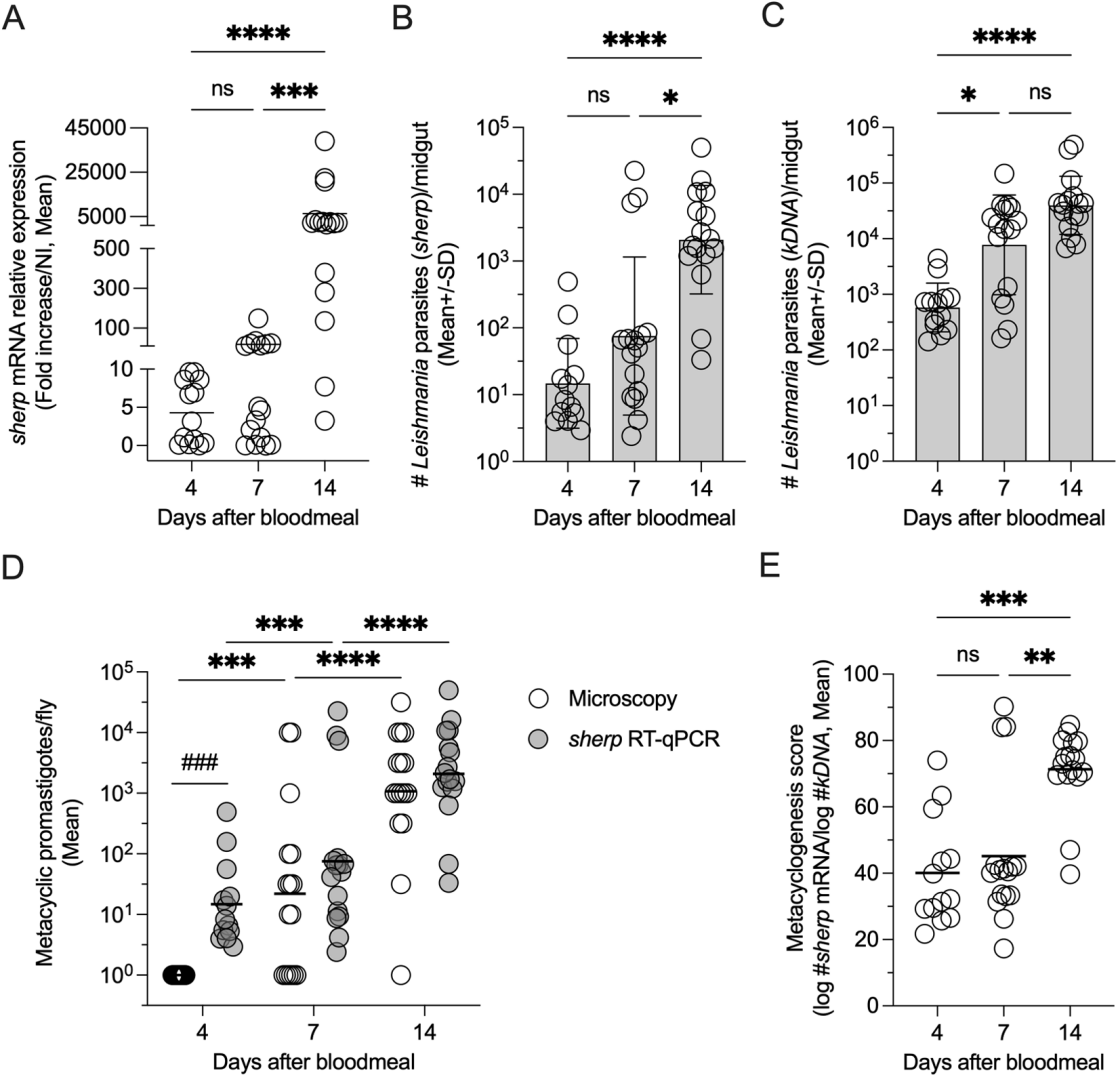
Detection and quantification of *Leishmania* metacyclic promastigotes in infected *Lu. longipalpis* sand fly midguts employing the *sherp* RT-qPCR. (A) Relative expression levels of the *sherp* gene determined by RT-qPCR at early (day 4-), mid (day 7-), and late (day 14-) time points post-sand fly infection (p.i.) of dissected infected *Lu. longipalpis* midguts normalized to *Leishmania*-specific 18S gene expression and expressed as a fold increase over uninfected midguts (calibrator samples). (B) Total number of parasites per infected midgut calculated employing the *sherp*-RT-qPCR. (C) Total number of parasites per infected midgut calculated employing the *kDNA*-RT-qPCR. (D) Total number of metacyclic promastigotes per infected midgut as determined by microscopic examination versus parasite number as determined by *sherp*-RT-qPCR from the same fly and shown in (B). (E) Calculation of a metacyclogenesis score using the *log* transformed number of parasites in matched samples determined by the *sherp* divided by the number determined by the *kDNA* RT-qPCR. Each symbol represents 1 dissected sand fly midgut. Asterisks indicate values that are statistically significant using a non-parametric Kruskall-Wallis test and a Dunn’s multiple comparison post-test (Panels A-C, E) or Multiple Unpaired t-tests with Welch correction (Panel D) comparing microscopy versus *sherp* PCR.

High numbers and frequencies of metacyclic promastigotes within infected sand flies are strong predictors of transmission potential and subsequent disease severity [12,,13]. Therefore, employing a *sherp*-RT-qPCR assay as a readout of both the presence of parasites and the degree of metacyclogenesis in sand flies would be a powerful molecular tool to identify vector species permissive to metacyclogenesis, determine the quality of sand fly infections in both laboratory-reared and field-caught flies, and identify hotspots of potential transmission in endemic areas. To determine if our *sherp*-RT-qPCR could detect *sherp* transcripts within sand flies, *Lu*. *longipalpis* sand flies were infected with *L*. (*L*.) *major* under laboratory conditions and maintained until the infections had undergone metacyclogenesis, expected to take approximately 14 days [14,33]. Infected flies were dissected at early (day 4-), mid- (day 7-), and late (day 14-) time points post-infection, and the midguts were processed and subjected to RT-qPCR (Fig 2A–D) or microscopic enumeration of metacyclic promastigotes (Fig 2D). The data was then analyzed and expressed as relative *sherp* gene expression (Fig 2A), number of parasites as determined by *sherp* (Fig 2B and 2D) or *kDNA* (Fig 2C) RT-qPCR, or the number of metacyclic promastigotes as determined by microscopy (Fig 2D). At day 4 p.i. we found low levels of *sherp* transcripts and parasite numbers based on *sherp* mRNA expression, as expected, given that the population of parasites at this time point is in the nectomonad stage of development. We also found no metacyclic promastigotes via microscopic examination on day 4 p.i. (Fig 2D). Parasites at this stage have successfully survived bloodmeal digestion and defecation and will colonize the sand fly gut for onward infection, amplification, metacyclogenesis, and subsequent transmission [14–15,33]. While it appears that metacyclogenesis is initiated sometime before day 7 post-infection (p.i.), as higher numbers (>100) of *sherp*-enriched parasites were already detected in a few dissected midguts on days 4 and 7 p.i. (Fig 2B), the dramatic and significant increase in relative *sherp* expression (approximately 600-fold) and number of parasites based on *sherp* expression (approximately 30-fold) between days 7 and 14 p.i (Fig 2A and 2B) versus a much lower (approximately 5-fold) and non-significant increase in the total number of parasites (Fig 2C) per midgut suggests the vast majority of metacyclogenesis occurs between days 7 and 14. In agreement with the results of the RT-qPCR, microscopic examination also showed metacyclogenesis increased over time and that both days 7 and 14 had matured infections with metacyclic promastigotes, which increased dramatically in number on day 14 p.i. (Fig 2D). Determination of the number of metacyclic promastigotes by microscopy versus parasite number by *sherp* RT-qPCR resulted in similar numbers of parasites at day 7 and 14 p.i. (Fig 2D, p≥0.339), confirming the ability of the *sherp* RT-qPCR assay to identify metacyclics at later time points. In contrast, analysis on day 4 revealed that the *sherp* RT-qPCR detected parasites but that these parasites were either not metacyclics or were below the detection limit of microscopy employing a haemocytometer. Importantly, to determine the degree of metacyclogenesis as a predictor of transmission potential, we also calculated a ‘metacyclogenesis score’ employing the *sherp* and *kDNA* RT-qPCRs (Fig 2E). A metacyclogenesis score of approximately ≥70 correlated with high numbers of parasites as determined by the *sherp* RT-qPCR and also revealed a robust increase in metacyclogenesis between days 7 and 14 (Fig 2E).

Of interest, at day 14 p.i. some midguts maintained low numbers of *sherp* transcripts despite high numbers of parasites as determined by *kDNA* RT-qPCR, suggesting “metacyclic-poor” flies exist within the infected sand fly population. Previous observations would indicate that these flies either do not transmit productive infections or transmit poorly, despite harboring established infections that have had sufficient time to expand and mature in the sand fly midgut [14–15]. Because the *sherp*-RT-qPCR indicates whether or not infected flies harbor mature infections based on the presence of metacyclic promastigotes, the information generated by the assay can be employed to predict if infected flies are likely to transmit the parasite and cause disease. Therefore, the assay could be effectively employed as a robust tool for monitoring transmission potential and vector competence and provide critical information for monitoring potential hotspots and intervention strategies in endemic areas.

### Evaluation of RT-qPCR for screening preserved whole sand flies as a potential tool for pathogen monitoring and transmission potential

**Fig 3:**
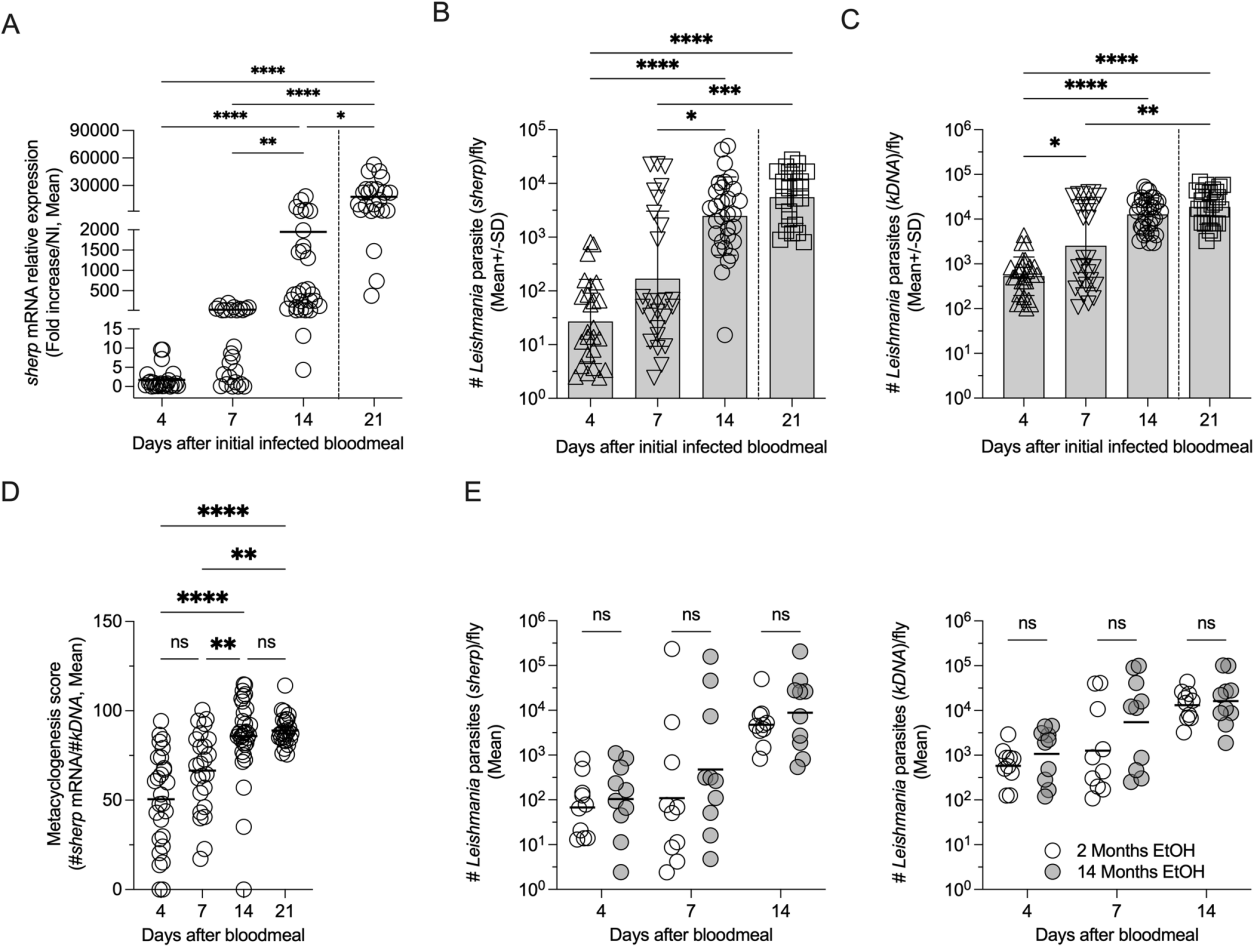
Screening of experimentally infected ethanol-fixed whole *Lu. longipalpis* sand fly samples for *sherp* gene expression or *kDNA* by RT-qPCR. (A) Expression levels of the *sherp* gene from ethanol-fixed sand fly samples on days 4-, 7-, 14-, and 21 p.i. The day 21 samples were flies used for experimental parasite transmission to naïve mice on day 14 p.i. (see Fig 4), as indicated by the dotted line. (B and C) The total number of parasites per infected sand fly employing the *sherp*-RT-qPCR (B) or *kDNA*-RT-qPCR (C). (D) Calculation of a metacyclogenesis score using the *log* transformed number of parasites in matched samples determined by the *sherp* divided by the number determined by the *kDNA* RT-qPCR. (E) Comparison of sample preservation in absolute ethanol medium at room temperature for 2- versus 14- months employing the *sherp* (left panel) or *kDNA* (right panel) RT-qPCR. Asterisks indicate values that are statistically significant using a non-parametric Kruskall-Wallis test and a Dunn’s multiple comparison post-test (Panels A-D) or Multiple Unpaired t-tests with Welch correction (Panel E) comparing 2- versus 14- months. In panels A-C, only significant differences are shown.

The ability to employ a detection method that allows for large-scale preservation of field-caught sand fly samples for subsequent analysis is likely to aid in data collection for vector-based surveillance programs in endemic areas. Currently, the microscopic analysis of different life-cycle stages requires either on-site analysis of live vectors or cryopreservation. Storage of samples for downstream RNA-based insect vector surveillance is also problematic when access to cryogenic materials or refrigeration is not available, posing a limiting factor to studies in resource-limited settings. To evaluate the field utility of the developed *sherp*-RT-qPCR assay, we applied the technique to whole sand flies (*Lu*. *longipalpis*) experimentally infected with *L*. (*L*.) *major* [14,33] and preserved using absolute ethanol, an economical and accessible means of preservation. Whole flies were collected at 4-, 7-, 14-, and after feeding on mouse ears on d14, 21-days p.i. Flies were preserved in absolute ethanol and stored at room temperature (either 2-4 months, panels A-D, or 14 months, panel E only, depending on the experimental group) until used for *sherp-* and *kDNA-* RT-qPCR analysis to assess their infections (Fig 3). Analysis at d21 was performed to investigate the impact of blood feeding on sand fly infections as previous observations have demonstrated that a second blood meal in flies with retained parasites can result in larger infections with a higher proportion of infective metacyclic promastigotes, suggesting infected flies can maintain high numbers of metacyclics and remain infectious over sequential blood meals [12]. The acquisition of the second blood meal in our experimental workflow occurred during the transmission of parasites to the mouse dermis. We once again observed a progressive increase in relative *sherp* gene expression from days 7 to 14 in preserved whole flies (Fig 3A), and this was also seen in the total number of parasites per sand fly as determined by *sherp* RT-qPCR (Fig 3B), reflecting the expected increases in metacyclics over the time course of infection. The detection of infection and the parasite loads in ethanol-preserved flies was confirmed by the *kDNA*-RT-qPCR (Fig 3C), demonstrating that ethanol preservation is a viable methodology to preserve infected sand flies for downstream analysis by RT-qPCR. Given the highly sensitive nature of the *kDNA*-RT-qPCR, we found a slight increase in the number of infected flies as a proportion of the total number of flies screened employing the *kDNA* versus *sherp* RT-qPCR, although this difference was negligible (Table 2). Furthermore, to evaluate the potential for flies with retained metacyclics to trigger metacyclic amplification after a second blood meal [12], day 14 p.i. flies were allowed to feed on mouse ears and acquire a second blood meal and were kept for an additional 7 days (21 total days post-initial infection). Screening the 21-day-old flies by RT-qPCR, we observed that the flies had a significantly higher relative expression of *sherp* transcripts versus day 14 p.i. (Fig 3A) and maintained high numbers of parasites per fly (Fig 3B, and 3C). However, we did not observe significant changes in the number of parasites as determined by *sherp* (Fig 3B) or *kDNA-* (Fig 3C) RT-qPCR between days 14 and 21. Similar results were found when employing the metacyclogenesis score (Fig 3D). These results demonstrate that infected sand flies likely remain infectious during sequential blood feeds, as previously suggested [12,15].

**Table 2:** Summary of sand flies screened by *sherp*- and *kDNA*-RT-qPCR.

Sample	Days after blood meal	No. screened	*sherp* - RT-qPCR	*kDNA* - RT-qPCR
No. infected (%)*	No. infected (%)
Artificially infected whole sand flies	4	30	25 (83.3)	28 (93.3)
	7	30	26 (86.7)	27 (90.0)
	14	35	33 (94.3)	33 (94.3)
	21^a^	23	23 (100.0)	23 (100.0)

*All *Leishmania*-infected whole flies detected by *sherp*-RT-qPCR were positive by *kDNA* -RT-qPCR.

^a^The flies used for the experimental parasite transmission to naïve mice at day 14 p.i. and obtained a second blood meal during the transmission, maintained for an additional 7 days (total 21 days post-infection, from the initial artificial infected blood-meal intake).

Optimal preservation of field-collected insect vector samples and stabilization of pathogen-derived RNA for downstream analysis is crucial for the implementation of RNA-based detection methodologies. To further evaluate whether long-term sand fly ethanol preservation at room temperature might affect the stability and reliability of the use of ethanol-preserved sand flies in field settings, we compared the detection of parasites in flies obtained from the same *Leishmania*-infected fly population stored in absolute ethanol at different time intervals; 2-months versus 14-months, at room temperature. All *Leishmania*-infected whole flies detected by *sherp*-RT-qPCR were positive by *kDNA* -RT-qPCR in both 2- and 14- months storage periods (Fig 3E) and we observed no significant differences in the ability to detect and quantify parasite load in flies using these different storage durations in absolute ethanol (Fig 3E), suggesting that long-term storage did not compromise the detection of parasites employing either RT-qPCR.

### Determination of transmission potential employing detection and quantification of *L.* (*L*.) *major* parasites in infected mice employing *sherp-* or *kDNA-*RT-qPCR

**Fig 4:**
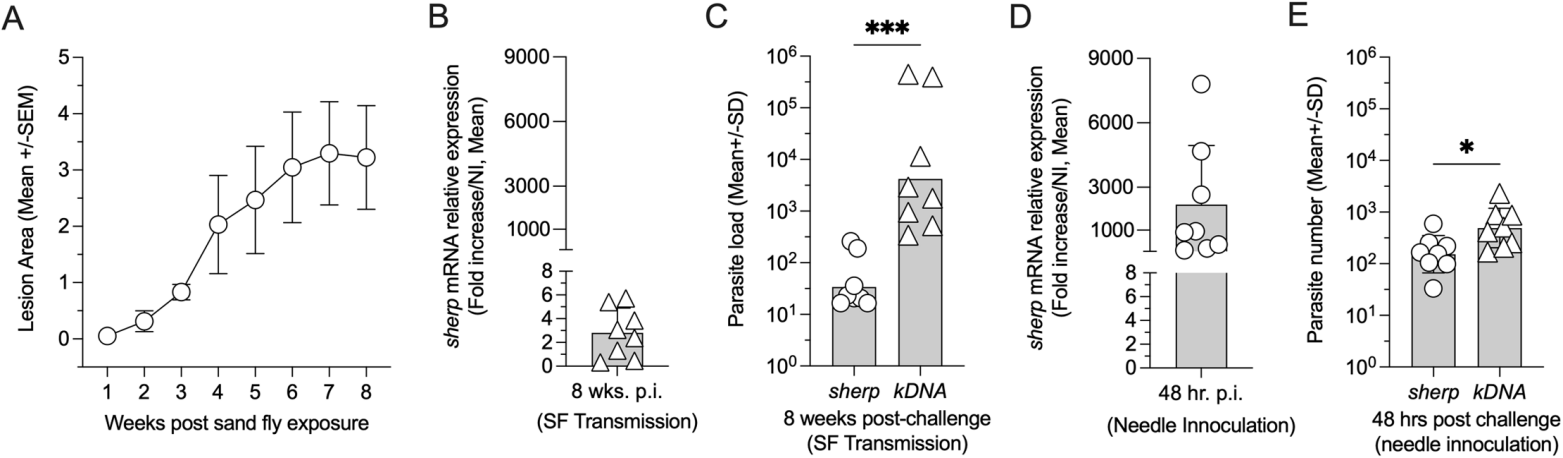
Detection and quantification of *L.* (L.) major parasites in infected mice employing sherp- or kDNA-RT-qPCR. Naïve female C57BL/6J (WT) mice were needled inoculated with 2 x 10^5^ *L.* **(*L*.)** *major* in the ear dermis (48 hr. data) or exposed to the bites of *L. major* infected *Lu. longipalpis* sand flies (8-week data, see Materials and Methods). (A) Mean ear lesion area (mm^2^) following infected sand fly exposure, n=8 ears. (**B and D)** *sherp* mRNA relative gene expression levels determined by RT-qPCR in ears at 8 weeks (B) and 48 hours (D) p.i., n=8 ears/group. (C and E) The total number of parasites per infected sand fly calculated employing the *sherp*- or total number of parasites per infected sand fly calculated employing the *kDNA*- RT-qPCR at 8 wks (C) or 48 hrs. (E) p.i.. Asterisks indicate statistically significant values, *P=0.014 and ***p=0.0002, n=8, employing a Mann-Whitney test.

On day 14 post-infection, the majority of whole flies contained high numbers of parasites as determined by the *sherp*-RT-qPCR and a high metacyclogenesis score (Fig 3B and 3D). To confirm that the increased relative expression and number of parasites as determined by the *sherp*-RT-qPCR, as well as the high metacyclogenesis score on day 14 p.i. correlated with *Leishmania* transmission and disease, we exposed anesthetized C57BL/6J (WT) naïve mice ears to the bites of the infected flies reported in Fig 3 on day 14 p.i. Animals were monitored every week to follow the development of lesions. As early as 2 weeks post-exposure, the animals developed cutaneous leishmaniasis ear lesions, which increased over the course of the experiment in 8/8 exposed ears (Fig 4A). At 8 weeks post-transmission, the animals were euthanized, ears processed, and used for *Leishmania* detection and quantification by the *sherp*- and *kDNA-*RT-qPCR (Fig 4B and 4C). In the mammalian skin, parasites exist in the intracellular amastigote lifecycle stage and are expected to have low to no *sherp* gene expression. As expected, analysis of the relative expression of *sherp* mRNA at 8 weeks post-transmission revealed very low to no relative expression (Fig 4B) and a correspondingly low number of parasites compared to the number of parasites calculated employing the *kDNA*-RT-qPCR (Fig 4C). The detection of parasites by *kDNA*-RT-qPCR also confirmed the successful transmission of parasites. The very low detection of *sherp* mRNA in the skin at 8 weeks p.i. is likely due to low *sherp* gene expression in amastigotes, as previously suggested [15]. In contrast, the detection of *Leishmania* by *sherp-* or *kDNA*-RT-qPCR at early time points following infection with metacyclic promastigotes revealed high levels of relative expression (compare Fig 4D with 4B) and, albeit significant, only a small difference in the total number of parasites detected by either primer set (Fig 4E), similar to previous observations [15] and suggests that *sherp* mRNA is still present in parasites 48 hours after inoculation into the skin.

## Discussion

Monitoring the transmission potential of insect vectors infected with vector-transmitted pathogens is an important epidemiological tool for predicting the risk and expansion of vector-transmitted diseases. In the case of the vector-borne parasitic disease Leishmaniasis, the frequency or number of metacyclic promastigotes in infected sand flies is a reliable predictor of successful transmission following the acquisition of a blood meal; this prediction is best made employing assays that reliably identify metacyclic-containing sand flies. The classical method of evaluation of *Leishmania* infection in sand flies is microscopical dissection and examination of fly midguts. However, it is a laborious and time-consuming procedure, typically needs to take place in the field, and is prone to discordant results depending upon the individual performing the differential life-cycle stage counts of parasite-containing sand flies. It is also inefficient to apply, especially in low-parasite burden foci, where one has to dissect large numbers of flies to confirm the presence or absence of the parasite. Thereby, molecular methods have been developed in the last three decades for the rapid detection and quantification of parasite genetic material in different biological samples [15,38–41]. In the present study, we sought to evaluate the utility of *sherp* RNA as a potential marker of viable *Leishmania* metacyclic abundance estimation, vector competence, and transmission potential in experimentally infected sand flies through a SYBR-Green-based *sherp*-RT-qPCR.

In nature, sand flies are likely to be infected with heterogeneous *Leishmania* forms (non-metacyclic and metacyclic promastigotes) and varying doses of parasites. The interaction of the *Leishmania* parasite along the fly gut depends on several intrinsic factors such as sand fly immunity, gut microbiota, antimicrobial factors, parasite virulence, blood/sugar meal, and environmental conditions, which influence parasite development and expansion during metacyclogenesis and subsequent transmission [8,9,14]. Metacyclic promastigotes are the end product of promastigote development within the sand fly vector [42], distinguished by morphology (rapid-swimming forms with an elongated flagellum) and high infectivity [43]. In addition to morphological differences, metacyclogenesis can also lead to changes in the expression of certain genes like *sherp* that are associated with the late-stage and infectious metacyclic form of the parasite [5,15,19–21,23,24].

Although *Leishmania* DNA amplification and parasite load quantification (qPCR) have been beneficial in insect vector surveillance, there has been a growing interest in developing an assay that can reliably quantify the abundance of *Leishmania* metacyclic promastigotes within infected sand flies as a potential tool for assessing/monitoring transmission dynamics in leishmaniasis endemic and surrounding areas. Previous studies have demonstrated that high frequencies or numbers of metacyclics per sand fly, not simply whether parasites are present, correlate with increased transmission frequency and subsequent disease severity [14,15]. By analyzing *sherp* mRNA transcripts, we observed the upregulation of *sherp* in dissected sand fly midguts and whole sand flies with mature infections, as well as in culture-derived purified metacyclics. The *sherp-*RT-qPCR consistently amplified *sherp* gene transcripts with high copy numbers from flies with mature infections as indicated by a high metacyclogenesis score, and these flies successfully transasmitted the disease. The ability to identify flies with mature infections based on the *sherp*-RT-qPCR assay is much more informative for predicting transmission potential versus simply determining if the parasite is present or not. Furthermore, storage of whole fly samples in absolute ethanol at room temperature for 2 or 14 months yielded sufficient RNA extracts for downstream RNA analysis by real-time PCR, demonstrating the feasibility of the methodology in low-resource field settings. Previous studies have also shown the utility of absolute ethanol as a vector storage medium for downstream DNA analysis like PCR, qPCR, DNA sequencing, and loop-mediated isothermal amplification (LAMP) [26,27,38,40,41,44]. In addition, it has also been shown previously that mosquito samples stored in absolute ethanol at 28 ^◦^C for 4 weeks before RNA extraction maintained fragment sizes over 800 bp and had similar RNA concentration as commercial RNA storage solution (Allprotect Tissue Reagent and RNAlater) and suggested that RNA isolated from absolute ethanol-preserved samples is sufficient to conduct downstream RNA analysis for real-time PCR or RNA-Seq [45]. We also observed unchanged detection sensitivity of *sherp*-RT-qPCR and amplification consistency of the target *Leishmania* RNA from infected flies stored in absolute ethanol at room temperature for months, indicating the stability of the target RNA over long-term storage. Detection of metacyclics employing ethanol-preserved whole infected sand flies for the first time in the present study represents an advantage over direct-microscopic quantification of sand fly-derived parasites as it does not require refrigeration of field-caught flies, on-site analysis, or dissection of sand fly midguts, a tedious and time-consuming process. Therefore, absolute ethanol may provide a viable economical alternative for sample preservation that can be utilized in resource-limited settings.

Studies have shown that multiple blood meals resulted in larger infections with a higher proportion of metacyclics and an increase in lesion frequency in mice bitten by twice-fed *Leishmania*-infected flies compared to single-fed flies [12]. It has also been suggested that multiple blood meals are likely to result in a higher proportion of high-dose transmitting sand flies [15]. In our present study, we observed a significant increase in the relative expression of *sherp* mRNA on day 21 p.i. in flies that obtained a second blood meal versus flies from the same population on day 14, prior to the second blood meal. However, no significant changes were observed in the number of parasites per fly on day 21 compared to day 14 p.i. employing either RT-qPCR assay. While the deposition of some parasites into the skin during the second blood meal on day 14 might partially explain the lack of a significant increase in parasite numbers observed on day 21 p.i., it is also possible that the numerical increase observed by others [12] may not occur under all conditions, as also suggested by studies which observed no significant differences in metacyclic numbers in *L*. (*L*.) *major*-infected *Phlebotomus duboscqi* employing multiple feeding conditions [46]. In our analysis of twice blood-fed flies on day 21 p.i., an appropriate control would have been flies that did not take a second blood meal. However, flies in this group demonstrated significant mortality, often resulting in no or only a few (<4) flies for analysis, as might be expected given the long time frame of the experiment. Without this control, a firm conclusion cannot be made regarding the importance of a 2^nd^ bloodmeal to maintain the high numbers of metacyclics observed at day 21, but we can conclude that a second blood meal does not diminish the infection, that flies are likely to remain infections following subsequent feeding events, and that, at least in terms of relative expression of *sherp* mRNA, an enhancement of metacyclogenesis does occur. Together with previously reported observations, the potential for enhanced high-dose/infection, metacyclic-rich fly transmitters following multiple blood meals may have epidemiological implications for leishmaniasis (“likely infection super-spreaders”) in endemic areas [15,30]. It is important to note that we observed the *L*. (*L*.) *major*-infected flies (“metacyclic-rich,” as expressed by *sherp* transcript) are able to transmit cutaneous leishmaniasis to a new naïve host on day 14 post-sand fly infection. Therefore, in this proof-of-concept study, the developed *sherp*-RT-qPCR would allow for an immediate real-time assessment of the presence of mammal-infective metacyclic promastigotes, thereby informing a determination of the potential for transmission in endemic communities.

Although *sherp* mRNA is detectable in parasite lifecycle stages other than metacyclics [5,15,19–21,23,24], the strong expression of *sherp* mRNA in sand flies harboring higher numbers of transmissible infective metacyclics versus flies containing no metacyclics makes *sherp* mRNA expression a good indicator of metacyclogenesis, a known predictor of increased transmission potential. While it is important to note that *sherp* expression is not metacyclic specific [5,15], and this should be viewed as a potential limitation of the assay at early time points in infection, this limitation does not preclude the usefulness of employing *sherp*-RT-qPCR in the analysis of field caught or experimentally infected flies versus simply quantifying total parasite load, as the latter is likely to change with different field and experimental conditions and provides limited information on transmission potential. We were also able to use the *sherp* and *kDNA* RT-qPCR assays to calculate a metacyclogenesis score that increased dramatically between days 7 and 14 and correlated with the increase in metacyclics as determined by microscopic counts and successful transmission, suggesting that the *sherp* RT-qPCR can both quantify parasites and determine the degree of metacyclogenesis. In addition, our *sherp*-RT-qPCR, which employs a novel set of primers, detected *sherp* expression in *L.*(*L*.) *major, L.* (*L*.) *infantum,* and *L.* (*L*.) *donavani* metacyclic promastigotes and could identify infected flies harboring mature infections following low-cost preservation in EtOH and without the need for time-consuming and skilled, labour-intensive microscopic dissection. All of these properties are highly advantageous in both field and experimental settings. Hence, not only will the developed RT-qPCR assay be useful as a tool for prevalence mapping, intervention surveillance, and vector incrimination but also for determining vector competence, metacyclogenesis, and transmission potential, important factors that can predict the likelihood of transmission that may augment experimental modeling and global efforts in reducing Leishmaniasis. These results highlight the potential of *sherp*-RT-qPCR and the need to further evaluate the field utility of the assay, including screening pools of field-caught sand flies (important for mass screening) from Leishmaniasis endemic areas for the study of natural sand fly infections and *Leishmania* transmission potential.

Taken together, we report the development of a potential entomological infection monitoring tool based on SYBR-Green-*sherp*-RT-qPCR using experimentally infected sand flies. The assay could contribute to better surveillance of parasite transmission dynamics, vector implication, and evaluation of intervention programs. At best, it could also provide an efficient tool for identifying transmission hotspots and areas of re-emergence of infections.
